# Liver Stiffness Values to Predict Occurrence and Recurrence of Hepatocellular Carcinoma

**DOI:** 10.3390/life14030342

**Published:** 2024-03-06

**Authors:** Cristina Stasi, Stefano Brillanti

**Affiliations:** Department of Medical, Surgical and Neuroscience Sciences, University of Siena, 53100 Siena, Italy; stefano.brillanti@unisi.it

**Keywords:** tumor microenvironments, hepatocellular carcinoma, liver stiffness, cutoffs, HCC occurrence, recurrence, elastography, BMI, NASH

## Abstract

Globally, liver cancer is the third most frequent etiology of cancer death, with the rates of occurrence of both new cases and mortality estimated to increase. Given the availability of multiple treatments, interdisciplinary management of the patient is crucial. Moreover, the diagnostic assessment of patients with severe liver fibrosis is essential for the staging of HCC and liver cirrhosis and early diagnosis of HCC. In this context, non-invasive evaluation plays a critical role in identifying prognostic factors of clinical application for the surveillance of the occurrence or recurrence of HCC. The new frontiers of transient elastography have become a useful tool to assess the risk of HCC occurrence and recurrence. There has been a major increase in studies investigating the cutoff liver stiffness value that best predicts the need for monitoring for the onset of HCC. Therefore, this review discusses the new advances that have occurred in the last four years on HCC, highlighting the new frontiers of non-invasive evaluation of HCC subjects, with particular attention regarding the clinical application of liver stiffness assessment for de novo HCC and predicting recurrence in patients with chronic HCV achieving sustained virological response after treatment with direct antiviral agents.

## 1. Introduction

According to Global Cancer Statistics 2020 [1], hepatic cancer is the sixth most frequent cause of cancer and the third most frequent reason for cancer death.

**Figure 1:**
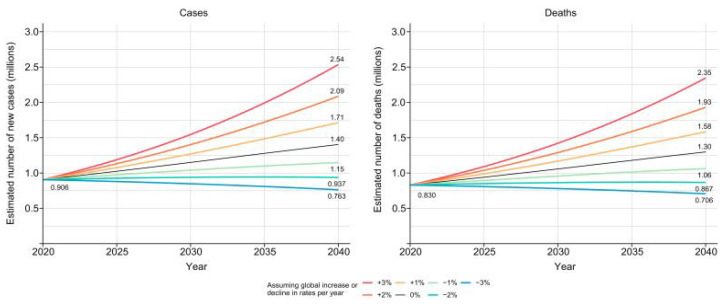
Prediction of HCC incidence and mortality. Rumgay et al. [2] predicted an increase in HCC incidence and mortality but depicted seven scenarios of increasing or decreasing rates by 3%, 2%, and 1% annually from the baseline year of 2020 to 2040. Source: Rumgay et al. [2]; License Number 5738260470253.

In 2020, approximately 905,700 hepatocellular carcinoma (HCC) diagnoses were made globally, and 830,200 subjects died. The global incidence rate of new cases of HCC was 9.5, and that of deaths was 8.7 per 100,000 subjects, with a higher incidence in Asia and North Africa. Hepatocellular carcinoma was one of the three main reasons for terminally ill cancer in 46 countries and was one of the top five reasons for terminally ill cancer in 90 countries. The incidence rates of new cases and mortality were lower in females than males in all countries [2]. To calculate the future number of primary liver cancer cases and deaths up to the year 2040, Rumgay et al. [2] used the current incidence and mortality for HCC at a global level, projecting it onto the United Nations medium-variant population, resulting in an increase in new HCC cases each year of 55.0% between 2020 and 2040. However, the majority of the increase in the number of cases and deaths was expected in countries with low Human Development Index values compared to countries with middle ones due to the major buildup in population and age; by far, the highest increment is expected in high-income countries, with an additional 55.7%, corresponding to 306,000 cases, and 57.6% deaths, corresponding to 302,000 deaths per year by 2040 (Figure 1) [2].

Liver carcinoma was considered among the leading causes of death due to oncological pathologies in Italy [3], and it ranked among the first countries for premature cancer mortality (in 2020) [1]. The interplay between genetic and immune factors, associated with a cellular microenvironment and viruses, plays a significant role in liver carcinogenesis [4]. Almost 90% of HCC cases develop in the context of an advanced stage of liver fibrosis/cirrhosis [5]. For this reason, the patient’s prognosis is a consequence of the stage of the tumor, concomitant diseases, and liver function. Moreover, the presence and severity of cirrhosis strongly influence the therapeutic choice. Antiviral treatments in the case of viral hepatitis and actions aimed at modifying the patient’s lifestyle in the case of liver disease due to alcohol abuse or metabolic dysfunctions play a significant role in the prognosis. As recommended by Italian guidelines, a multidisciplinary discussion is needed to avoid over- or under-treatment, particularly in patients with intermediate-stage (multinodular tumor localized in the liver and with good liver function) or locally advanced HCC (intra-hepatic vascular invasion) that can be treated with approaches ranging from liver transplantation to systemic, in consideration of the wide variability in the degree of intrahepatic spread of HCC [6].

Currently, several lines of treatment are available, such as surgical therapies (liver transplant and resection), radiotherapy, and ablative and systemic therapies, which find their indication in different stages of HCC and cirrhosis, making the diagnostic process of fundamental importance for both the staging of HCC and liver cirrhosis and for early detection of HCC [6].

Based on these premises, this review discusses the new advances that have occurred in the last four years regarding HCC, highlighting the new frontiers of non-invasive evaluation of HCC patients, with particular attention focused on the clinical application of liver stiffness assessment for de novo HCC and predicting recurrence in chronic HCV subjects, achieving sustained virological response (SVR) after treatment with direct-acting antivirals (DAA).

## 2. Role of Liver Inflammation and Fibrosis in Hepatocarcinogenesis

The interactions between continuous inflammation, exaggerated production of reactive oxygen species (ROS), and dysregulation of cell–cell communication are involved in the occurrence/recurrence of HCC in different ways [4]. In addition, immunosuppression, mutagens, and metabolism work synergically with oncogenic viruses to stimulate cancer development [7]. HCV has a different manner of HBV concerning the induction of cancerogenesis, which is mediated by the interaction between the viral NS5A protein and cellular components. The consequences of this interaction result in a shift toward viral protein synthesis instead of cellular and lipogenesis, causing an accumulation of free fatty acid proteins in the cytosolic space, which, in turn, by oxidative stress, leads to the activation of the NFkB pathway [5].

Chronic inflammation generally results from disequilibrium between extracellular matrix (ECM) production and its destruction [8]. The accumulation of ECM and chronic inflammation are identified as a source of the potent damage-associated molecular pattern (DAMP) [9], leading to dysregulation of immune cells, which in turn increases the ROS and proinflammatory cytokines release into the liver and ultimately also results in modifying its microenvironment, particularly during chronic viral infections [10]. Liver fibrosis is a reversible process caused by chronic liver damage and is involved in HCC development. Extracellular matrix production is mainly due to hepatic stellate cells (HSCs) that, during liver injury, cross a phenotypical transformation into activated myofibroblast-like cells, synthesizing proinflammatory/proangiogenic cytokines and a large amount of ECM components [8]. It was demonstrated that fibroblasts found in the tumor environment called carcinoma-associated fibroblasts (CAFs) specifically express smooth muscle actin, EGF, HGF, IGF-1 and -2, and matrix remodeling enzymes such as MMPs, demonstrating the importance of tumor stroma, and CAFs in particular, to the process of tumor progression [11]. New evidence underlined the involvement of platelets in hepatocarcinogenesis. After their activation, the platelets seem to contribute in different ways to promote HCC development. In fact, on the one hand, the release of α-granules and dense granules containing inflammatory cytokines, chemokines, and growth factors stimulates tumor development; on the other hand, secretion of proangiogenic factors (VEGF-A and FGF) and interaction with endothelial cells via CD40L [12,13], P-selectin, and GP Iib-IIIa [14] induce the angiogenic switch. Moreover, platelet microparticles promote the switch from antitumor macrophages towards a pro-tumoral phenotype [15]. Furthermore, both direct contact and release of TGFβ can affect the cytotoxic potential of natural killer (NK) cells, with a subsequent immunosuppressive microenvironment facilitating tumor growth [16].

## 3. New Frontiers of Non-Invasive Evaluation of Patients with Hepatocellular Carcinoma: Elastography

The nodal point of chronic hepatopathies in general, and those of viral origin in particular, is represented by their ability to undergo fibrotic progression in a dynamic context in which accumulation of ECM is associated with constant degradation and remodeling of the matrix itself. On the one hand, this “balanced” process can evolve towards cirrhosis, but, on the other hand, it can retrace the reverse path. Although theoretically possible after removing the causal agent, this reversibility is still a discussion topic regarding the natural history of cirrhosis. The precise distinction between absent/non-significant fibrosis and advanced fibrosis and cirrhosis is crucial for both diagnostic and therapeutic strategies and in terms of monitoring chronic HCV patients after DAAs or chronic HBV patients treated with nucleosides/nucleotide analogues. Over the past fifteen years, several non-invasive methods have been proposed to predict the presence of fibrosis in chronic hepatopathies [17,18]. The non-invasive evaluation of hepatic fibrosis has represented considerable advancement in clinical hepatology, especially in staging viral hepatitis.

These methods have almost totally replaced hepatic biopsy and can evaluate with appropriate scoring systems not only the degree of hepatic fibrosis and necro-inflammatory activity but provide static information on the process of liver fibrogenesis; therefore, they do not allow for obtaining data on the evolution of the tissue repair phenomenon or the speed of progression towards cirrhosis. To be diagnostic, a liver biopsy needs to involve a minimum of 11 complete portal spaces, and it must be long, at least 2 cm, and 1.4 mm thick regardless of the route through which it is carried out (percutaneous, laparoscopic, surgical, or transjugular) [17]. However, a hepatic biopsy is characterized by invasiveness and low compliance by patients, the possibility of life-threatening complications, sampling errors, and observer-dependent diagnostic variability [17,19]. Most non-invasive tools are based on algorithms, including biochemical parameters, which provide proven diagnostic accuracy at the beginning and end of the fibrogenic evolution. Serum markers for the prediction of the fibrosis stage are divided into two groups: (1) “direct markers” that reflect the spread in the systemic circulation of peptides involved in the accumulation of extracellular fibrillar matrix (fibrogenesis) or involved in degradation (fibrolysis), or more generally involved in tissue inflammation, and (2) “indirect markers”, a combination of clinical parameters and biochemical changes found in chronic hepatitis. The predictive ability of possible combinations of these markers is provided by their inclusion in a mathematical algorithm [17,18]. 

In hepatology, the launch of elastography was characterized by initial skepticism. However, several studies, mainly conducted in chronic HCV patients, have indicated that this method represented the “missing tool” in hepatology, with an exponential increase in attentiveness. The elastography (Fibroscan^®^, Echosense, Paris, France) consists of a 3.5 MHz probe with a transducer mounted on the axis of a vibrator. A pediatric probe (5 mhz) and a probe for obese people have also been introduced. The method is simple, fast, and non-invasive, and patients are subjected to an examination with high compliance. The examination is performed on the right lobe of the liver through the intercostal spaces with the patient in dorsal decubitus and the right arm in maximum abduction [17,18]. The operator, assisted by A-Mode ultrasound images, selects a portion of the liver in the right hepatic lobe at least 6 cm thick and devoid of large vascular and bile structures. At least 60% of the measurements, taken on a 1 cm in diameter and 4 cm long liver tissue cylinder, a volume at least 100 times larger than a biopsy sample, represent the measure of liver stiffness. The final result of the examination is expressed as the median of the valid measurements and, for good quality of measurements, from the interquartile range (IQR), which must be less than 30% of the median value. Stiffness measurement cannot be performed in patients with ascites as the elastic wave does not propagate through liquids [17], while confounding factors are flares of transaminases and meals. Elastography is characterized by high intra- and inter-observer repeatability (0.98) [20,21,22].

However, inter- and especially intra-observer repeatability are influenced by variables such as body mass index (BMI) (particularly when 28) [22], and, therefore, LSV should be used with caution in cohorts of patients who are either overweight or frankly obese. For this reason, an XL probe was introduced for obese patients [23,24].

The WHO [25] recommended BMI levels between 18.5 and 24.9 as normal values. Liu et al. [26] recently compared LSV across different BMI categories and found that subjects with normal BMI showed the lowest risk of having elevated LSV compared to those who were underweight and obese. Recently, controlled attenuation parameters have been used to study and quantify hepatic steatosis in patients with non-alcoholic fatty liver disease (NAFLD) [27] and non-alcoholic steatohepatitis (NASH) [28].

The association between tobacco use and diabetes is related to an increase in LSV and fibrosis [29].

At the same time, the role of regular consumption of alcoholic beverages could be overall “underestimated” in any hepatological patient. In a study dated 1992, even among the men who participated in the seven national studies [30] in Crevalcore and Montegiorgio, daily alcohol consumption was generally very high. Currently, the WHO believes that no level of alcohol is safe for health [31].

Studies conducted predominantly in viral hepatitis [32,33,34] suggested that transient elastography is a valuable method for diagnosing advanced fibrosis and cirrhosis and for ruling out significant fibrosis (> or = F2) [17]. Studies confirmed both the role of elastography as a diagnostic tool for liver stiffness evaluation as a “surrogate” of liver fibrosis and to reduce the number of liver biopsies in chronic hepatic diseases and drug-induced fibrosis [35] and as a means of monitoring the progression/regression of hepatic fibrosis [33,36], as well as for prediction of outcomes [37,38].

Several studies have proposed the usefulness of elastography for the cross-sectional and longitudinal assessment of fibrosis and for the baseline assessment of chronic HCV, especially regarding diagnosis of severe fibrosis and cirrhosis, which affects the post-therapy monitoring of these patients as well as the indication regarding the performance of gastroscopy for the detection of esophageal varices. According to the recommendations of Baveno VI, endoscopic screening is indicated in patients with platelets < 150,000 and stiffness > 20 kPa [39].

There are three main ultrasound-based elastographic techniques, and they differ in the approaches used: transient elastography (Vibration-Controlled Transient Elastography, TE) employs an external mechanical push; acoustic radiation force impulse methods employ an internal acoustic push; and the strain elastography (SE) technique employs tissue deformation (frame-by-frame differences) with strain, produced by push on the body surface or internal physiological movement. TE and ARFI are shear wave-based techniques measuring the speed of shear waves in tissues; for TE and ARFI, the calculated shear wave velocity, correlated to liver stiffness, can be converted to kilopascals [40]. These quasi-static elastography methods are more reproducible and quantitative because they rely on automatic shear wave generation. TE is the most widely used and validated technique and presents high performance and prognostic value for cirrhosis. This is the reason why this review focuses on the non-invasive assessment of HCC occurrence/recurrence by TE. Among the disadvantages of this technique, we cite the reduction in applicability in the case of ascites and the need for operator experience [41]. Moreover, the other limits of LSV by TE are represented by confounding factors such as elevated alanine aminotransferase (ALT) levels, extrahepatic cholestasis, congestive heart failure, excessive alcohol intake, and food intake. TE should be performed in fasting patients [41]. Overweight and other comorbidities, such as diabetes, can affect LSV as compared with normal-weight patients because they can contribute to an increase in inflammation or liver fibrosis. Diabetes in obese subjects increased the risk of LSV, corresponding to advanced fibrosis [42]. ARFI presents higher applicability than TE in the case of obesity. Magnetic resonance elastography (MRE) induces harmonic vibrations of acoustic-range frequencies in tissue and displays the propagation of these vibrations in tissue to calculate quantitative values for tissue mechanical parameters. The potential advantages of MRE are the ability to scan larger volumes of the liver (reducing error due to variability in fibrosis in the liver), higher applicability than TE (ascites and obesity), and high performance for cirrhosis [41]. The disadvantages are represented by the fact that this method is not applicable in case of iron overload, requires an MRI facility, and the cost of this procedure. MRE seems to be a promising technique capable of differentiating benign and malignant focal lesions without the use of an intravenous contrast agent [43]. The study by Venkatesh et al. [44] suggests that a cutoff value of 5.0 kPa may be very accurate (accuracy = 100%) for differentiating benign focal masses from malignant tumors.

Recently, elastography has been proposed as a valuable tool for assessing the risk of hepatocellular occurrence and recurrence.

A recent review by Marasco et al. [45] summarized the available literature on the evaluation of the different non-invasive tests specifically regarding FIB-4, platelet ratio index, and liver stiffness for predicting primary HCC occurrence. Many of these studies do not analyze the diagnostic accuracy in predicting HCC in terms of sensitivity and specificity depicted by the Receiver Operator Characteristic (ROC) curve.

Similarly, many of the studies conducted subsequently and reported in our review do not report analyses of diagnostic accuracy. Considering the studies conducted in HCV patients undergoing DAA therapy reported in the literature review by Marasco et al. [45], among the indirect serum markers of liver fibrosis, the AUROCs of the aspartate aminotransferase to platelet ratio index have a range between 0.870 [46] and 0.89 [47] in HCV patients treated treated with IFN-based treatment. The AUROCs of FIB-4 in the same type of patients reach values of 0.86 and 0.85 (Na et al. [47]). As regards the prediction of HCC, in the study by Izumi et al. [48], the AUROCs present a value of 0.806. Hepatitis B and C, non-alcoholic steatohepatitis, and alcohol abuse are differently associated with the progression of fibrosis. Therefore, different LSV cutoffs for staging liver fibrosis have been validated for these main etiological factors [41]. Moreover, LSV requires validation in different patient populations, such as metabolic dysfunction-associated steatotic liver disease (MASLD), autoimmune hepatitis, and primary sclerosing cholangitis, due to the limited single-etiology studies. It is for this reason that our literature review focuses on one of these main risk factors for progression to liver cirrhosis and the development of hepatocellular carcinoma.

Future research directions in non-invasive evaluation techniques for HCC are probably represented by the development of multiparametric approaches or the integration of imaging modalities. A very recent study [49] in a larger cohort of Asian patients with MASLD evaluated whether the combined assessment of LSV at baseline and its changes during the follow-up period prognosticates outcomes. Kobayashi et al. [49] found that baseline LSV was associated with decompensated cirrhosis, HCC, all liver-related events, extrahepatic malignancies, and all-cause mortality except cardiovascular mortality. Meanwhile, ΔLSV was significantly associated with decompensated cirrhosis, HCC, and it could predict the development of cirrhosis. An increase in LSV > 19% during follow-up was also associated with patients at high risk of outcomes such as HCC detected at MRE, suggesting that the ΔLSV could be used to select patients for closer monitoring with MRE.

## 4. Clinical Application of Liver Stiffness Assessment for Hepatocellular Carcinoma Occurrence Prediction

Several studies have demonstrated a strong association between liver stiffness values (LSV) and HCC development in the last decade. In particular, most of these studies were conducted after the advent of DAAs, a therapeutic regimen that represented a Copernican revolution in the treatment of HCV, also reaching a sustained virological response (SVR) > 95% in most complicated patients [50,51,52].

A recent study [53] evaluated the changes in LSV up to 96 weeks after SVR in 185 chronic HCV-infected patients treated with DAA, investigating the LSV cutoff associated with HCC development. This study found significant differences in LSV between baseline and 24, 48, 72, and 96 weeks after SVR. Despite the reduction in LSV after SVR, during the 41.6-month follow-up, the HCC occurrence rate was 1.17 per 100 person years, suggesting monitoring for complications, particularly in patients with LSV > 8 kPa at 48 weeks after SVR.

Pons et al. [54], in a 2-center prospective study, enrolled 572 compensated advanced chronic HCV patients, DAA-treated, achieving SVR and with LSV > 10 kPa at baseline. In these patients, the authors studied the incidence of liver-related events, particularly HCC, during a median follow-up of 2.9 years. HCC occurred in 25 patients (4.4%) at an incidence rate of 1.5/100 patient years, with a median time to HCC occurrence of 1 year. When the authors combined follow-up albumin and LSV to identify risk groups, they found that both albumin levels (<4.4 g/dL) and LSV (>20 kPa) can identify patients at the highest risk of presenting with HCC. Given that a zero-risk subpopulation cannot be found, these findings suggest HCC monitoring for all advanced chronic liver disease patients before therapy.

A retrospective cohort study [55] of 1850 veterans with HCV-related cirrhosis and SVR monitored the HCC occurrence over 5099 person years, after SVR until death or the end of the study, and evaluated if LSV after SVR is useful to stratify HCC risk. The adjusted annual risk of HCC was 2.03% for patients with LSV < 10 kPa after achieving SVR, 2.48% for those with LSV 10–14.9 kPa, 3.22% for LSV of 15–19.9 kPa, 5.07% for LSV of 20–24.9 kPa, and 5.44% for LSV > 25 kPa. Given that the adjusted HCC annual risk was <0.4% for patients with LSV < 5 kPa and without diabetes mellitus, the authors suggest discontinuing surveillance in this kind of patient.

Another study [56] on the veterans cohort identified patients with an existing diagnosis of HCV or NAFLD assessed with liver transient elastography from 2015 to 2019. In the 26,161 HCV group, there were 496 patients with chart-confirmed HCC. The incidence of HCC increased with LSV, with incidence rates of 0.28, 0.93/100,000 person years for LSV range < 9.5 kPa, 1.28 for LSV between 12.5 and 14.5 kPa, and 2.79 for LSV > 14.5 kPa. Moreover, within a median follow-up of 2.3 years, the study demonstrated an increasing incidence of HCC in HCV patients, which was 0.82% per year with increasing LSV.

Ciancio et al. [57] identified the HCC incidence low-risk group in 1000 HCV patients with advanced chronic liver disease, achieving SVR by DAAs, and 90% of the patients with LSV between ≥9.5 and ≤14.5 kPa after a median follow-up of 4 years had an HCC occurrence risk < 1%/year. According to this study, Nakai et al. [58] retrospectively analyzed risk factors for de novo HCC in 567 patients who achieved SVR24 after IFN-free treatment in only cases with LSV < 8.4 kPa at the time of SVR24; the 4-year cumulative HCC incidence rate tended to be higher in patients aged > 71 years, suggesting that advanced age and progression of liver fibrosis by transient elastography are important risk factors for HCC after SVR. The study by Nakai et al. [58] analyzed the ROC curves to examine the best cutoff value for the occurrence of HCC according to pre-treatment and post-treatment liver stiffness. The results revealed that the pre-treatment cutoff value was 9.2 kPa (sensitivity, 68%; specificity, 81.5%), and the cutoff value at the time of SVR24 was 8.4 kPa (sensitivity, 76.5%; specificity, 73.3%).

On the other hand, Reinoso-Pereira et al. [59], in a prospective cohort of 99 Brazilian HCV patients with cirrhosis, demonstrated that an LSV > 21.1 kPa was the best cutoff and was independently associated with higher HCC development. Similarly, a baseline LSV > 20 KPa cutoff was found in an Italian cohort of cirrhotic HCV patients treated with DAAs [60] as an independent predictor of HCC.

Another study [61] compared patients with post-treatment LSV > 12.5 kPa, but not baseline LSV, with those with post-treatment LSV > 20 kPa, showing higher rates of developing decompensated cirrhosis and the composite outcomes of death, liver transplant, decompensated cirrhosis, or HCC.

Yoo et al. [62] compared the occurrence of HCC in HCV patients treated with DAA over three years with a historical cohort treated with pegylated interferon (peg-IFN). This study highlighted that the decrease in LSV was significantly larger in the DAA group than in the peg-IFN group at both 48 weeks and 96 weeks, but the HCC incidence was not significantly different between the DAA and peg-IFN groups (5.5% vs. 5.4%) at 144 weeks.

Alonso López et al. [63] identified patients at low HCC risk based on the prediction of non-invasive markers and their changes after SVR through an algorithm including transient elastography. The model predicted 0% of HCC occurrence at 3 years in patients with score 0 (baseline LSV > 17.3 kPa, albumin > 4.2 g/dL, and 1-year DeltaLSV > 25.5%) versus 5.2% in patients with scores of 1–3.

Considering the longitudinal evaluation of LSV, several studies demonstrated a parallel decrease in LSV and HCC occurrence risk.

Some clinical trials suggest the efficacy and utility of non-invasive evaluation methods in HCC management. A recently published clinical trial [64] evaluated LSV measured by two-dimensional shear wave elastography (2D-SWE) to predict post-hepatectomy liver failure in HCC patients (146) with a Child–Pugh score of 5, randomly divided into training (*n* = 97) and validation (*n* = 49) groups. The study demonstrated that LSV was associated with the development of post-hepatectomy liver failure in HCC patients with a CP-5 score, with an AUC of 0.76 in the validation group.

A large clinical trial [65] conducted in 2876 subclinical cirrhotic HBV patients with LSV ≥ 13 kPa evaluated whether LSV can identify patients at increased risk of developing HCC. Subclinical cirrhosis identified by LSV has been shown to identify patients with chronic HBV infection at increased risk of developing HCC. A study [66], expected to conclude in late 2024, is underway in patients with liver cancer undergoing therapy to evaluate the prognostic significance of liver stiffness, the rate and severity of treatment complications, and the association with liver stiffness. Table 1 summarises studies on chronic HCV after DAA treatment reporting LSV in predicting hepatocellular carcinoma occurrence and recurrence.

## 5. Clinical Application of Liver Stiffness Assessment for Hepatocellular Carcinoma Recurrence Prediction

Different treatment options are currently available for the treatment of HCC, but a wide range of incidences of HCC recurrence burdens all of them.

Lee et al. [70] reported recurrent disease in 47.2% of 506 patients enrolled in the study, occurring within 9 months after hepatectomy. In a recent meta-analysis, Bzeizi et al. [71], regarding 1673 patients who met the Milan criteria before liver transplantation, found a pooled prevalence of HCC recurrence of 44% (95% CI: 18–24) in comparison with a pooled prevalence of 47 (95% CI: 37–57) patients (*n* = 511) who did not meet the Milan criteria. In another meta-analysis [72] conducted on nineteen cohort studies for a total of 2764 patients, the median 5-year survival rates after repeat hepatectomy (525 patients), ablation (658), and transarterial chemoembolization (TACE) (855) were 35.2, 48.3, and 15.5 percent, respectively.

Based on these data, finding non-invasive tools that can accurately predict the risk of HCC recurrence is crucial.

Shousha et al. [67] studied the impact of LSV and some components of tumorigenic cells, called cancer stem cells (CSCs), including epithelial cell adhesion molecule (EpCAM) and cytokeratin-19 (CK-19) markers, to predict HCC recurrence and their impact on the clinical outcome of HCC and overall survival. This study enrolled 179 patients with HCV-related HCC with 74.9% Child–Pugh class A and MELD scores of 10 (8–11). The most common treatments were TACE (79.8%), microwave ablation (15%), and hepatic resection (3.9%). Fifty-five patients developed HCC recurrence following successful complete ablation and were mainly treated with TACE. LSV in these patients significantly increased 6 months following ablation. The LSV and EpCAM levels proved to be important factors affecting overall survival. The study by Kim et al. [68], comparing the outcomes of cirrhotic and non-cirrhotic groups after liver resection in solitary HCV-related HCC patients, identified as risk factors for HCC recurrence, showed that non-invasive markers and the presence of cirrhosis (characterized by higher LSV compared to the non-cirrhotic group) were not related to HCC recurrence or death in multivariate analyses.

Similarly to what was demonstrated in other studies conducted in patients treated with IFN [20], DAA regimens were also associated with a reduction in LSV [32,69]. An interesting retrospective study [69] evaluated LSV variation as a predictor of HCC occurrence/recurrence risk after DAA treatment in cirrhotic HCV patients with a mean LSV at the baseline in the entire cohort (with and without HCC development after treatment) of 18 kPa and a median 15 months follow-up. To our knowledge, this study, for the first time, demonstrated a significantly lower LSV decrement (−18%) in patients with HCC development after DAA treatment compared with LSV (28.9%) regarding those without HCC occurrence. Moreover, a decrement in LSV lower than −30%, a Child–Turcotte–Pugh score of B, and a history of previous HCC were strongly associated with HCC occurrence/recurrence risk. Based on these findings, the authors suggest surveillance of these cirrhotic HCV patients, evaluating the LSV at EOT, and calculating the variations in LS to improve the HCC screening for HCC occurrence/recurrence after DAA treatment [69].

## 6. Conclusions

Following the advent of DAA, there was a major increase in studies investigating the role played by transient elastography for accurate prediction of HCC occurrence in patients with HCV-related advanced chronic liver disease before treatment. Most of the studies agree with an LSV cutoff > 8 kPa to indicate careful monitoring of these patients for the onset of outcomes. Overall, studies suggest that elevated LSV at baseline and delta stiffness during follow-up could be used as predictors of outcomes in these patients, suggesting closer follow-up. More studies are probably needed to establish an LVS cutoff strongly associated with HCC occurrence. Moreover, few studies have been conducted to determine the post-treatment LSV associated with a high risk of developing HCC. In parallel, future research should be directed toward longitudinal studies to identify the clinical application of LSV to predict HCC recurrence.

## Figures and Tables

**Table 1:** Studies on chronic HCV after DAA treatment reporting liver stiffness values (before and after treatment) in predicting hepatocellular carcinoma occurrence and recurrence.

Authors	Study Design	HCC and/or Pts Tot (*n*)	Follow-Up	Country	HCC	Cutoffs
Rodprasert et al. 2023 [53]	retrospective	8/185	48 weeks post-SVR	Thailand	1.17 per 100 person years (incidence rate)	≥8 kPa associated with poor liver-related outcomes, including HCC
Pons et al., 2023 [54]	prospective	25/572 (3% were lost to follow-up)	1 year after EOT	Spain	1.5 per 100 patient years	<10 kPa during follow-up, associated with low-risk HCC occurrence
John et al., 2023 [55]	retrospective	1850 in follow-up over 5099 person years	After SVR, until death, HCC, or end of study	US	- 2.03% (adjusted annual risk) - 2.48% - 3.22% - 5.07% - 5.44%	- in LSV < 10 kPa - in LSV 10–14.9 kPa - in LSV 15–19.9 kPa - in LSV 20–24.9 kPa - in LSV ≥ 25 kPa
Davitkov et al., 2023 [56]	retrospective	496/26,161	2.3 years (median)	US	- 0.28 (95% CI 0.24,0.34) - 0.93 (95% CI 0.72, 1.17) - 1.28 (95% CI 0.89, 1.79) - 2.79 (95% CI 2.47, 3.14)/100,000 person years (incidence rate)	- LSV < 9.5 kPa - LSV 9.5–12.5 kPa - LSV12.5–14.5 kPa - LSV > 14.5 kPa
Ciancio et al., 2023 [57]	prospective	71/1000	4 years (median)	Italy	HCC risk < 1%/year (incidence rate)	LSV ≥ 9.5 ≤ 14.5 kPa
Reinoso-Pereira et al., 2022 [59]	prospective	20/99	5 years (mean)	Brazil	5.54-fold higher chance of developing HCC (hazard ratio)	LSV > 21.1 kPa
Vutien et al., 2020 [61]	retrospective	20/877	27.3 months (mean) after treatment	US	death, liver transplant, decompensated cirrhosis, or HCC higher rates	LSV > 20 kPa
Yoo et al., 2022 [62]	prospective (Clinical trials registration: ClinicalTrials.gov (NCT02865369)	5/86 treated with DAA	48, 96, 144 weeks after starting DAA	Korea	6.1% HCC patients at 96 weeks in the DAA group	Baseline LSV = 17.6
Morisco et al., 2020 [60]	prospective	66/706	24 months after SVR confirmation	Italy	2.5 incidence rate	LSV > 20 kPa
Alonso López et al., 2020 [63]	prospective	35/993	45 months (median)	Spain	at 3 years in Pts with score 0 (baseline LSV ≤ 17.3 kPa, albumin > 4.2 g/dL, and 1-year DeltaLSV > 25.5%) HCC occurrence = 0%	LSV ≤ 17.3 kPa
Shousha et al., 2020 [67]	prospective	55 recurrences after procedure/179	6 months	Egypt	47.2% of all recurrences occurred after 9 months postoperatively	LSV increased 6 months post-ablation
Kim et al., 2022 [68]	prospective	31.9% in the non-cirrhotic group and 39.6% in the cirrhotic group/207	47 months	Korea/USA	absence of cirrhosis does not predict HCC recurrence	Presence of cirrhosis (characterized by a higher LSV) not associated with HCC.
Ravaioli et al. [69]	retrospective	13 HCC recurrences and 7 recurrences/139	15 months (median)	Italy	HCC occurrence/recurrence increasing risk in Pts with lower DeltaLSV	DeltaLSV > −30% associated with disease-free survival

Pts, patients; HCC, hepatocellular carcinoma; SVR, sustained virological response; kPa, kilopascal; LSV, liver stiffness values; DAA, direct-acting antivirals.

## Data Availability

Not applicable.
